# A Review on *Annona muricata* and Its Anticancer Activity

**DOI:** 10.3390/cancers14184539

**Published:** 2022-09-19

**Authors:** Suganya Ilango, Dipak Kumar Sahoo, Biswaranjan Paital, Kavibharathi Kathirvel, Jerrina Issac Gabriel, Kalyani Subramaniam, Priyanka Jayachandran, Rajendra Kumar Dash, Akshaya Kumar Hati, Tapas Ranjan Behera, Pragnyashree Mishra, Ramalingam Nirmaladevi

**Affiliations:** 1 Department of Biochemistry, Biotechnology and Bioinformatics, Avinashilingam Institute for Home Science and Higher Education for Women, Coimbatore 641043, India; 2 Department of Veterinary Clinical Sciences, College of Veterinary Medicine, Iowa State University, Ames, IA 50011, USA; 3 Redox Regulation Laboratory, Department of Zoology, College of Basic Science and Humanities, Odisha University of Agriculture and Technology, Bhubaneswar 751003, India; 4 Dr Abhin Chandra Homeopathic Medical College & Hospital, Bhubaneswar 751001, India; 5 Department of Community Medicine, SCB Medical College and Hospital, Mangalabag, Cuttack 753007, India; 6 Department of Floriculture & Landscaping, College of Horticulture, Odisha University of Agriculture and Technology, Chipilima, Sambalpur 768025, India

**Keywords:** *Annona muricata*, ethnomedicinal, bioactive metabolites, pharmacological activities, anticancer activity

## Abstract

**Simple Summary:**

Cancer is becoming more prevalent, raising concerns regarding how well current treatments work. Cancer patients frequently seek alternative treatments to surgery, chemotherapy, and radiation therapy. The use of medicinal plants in both preventative and curative healthcare is widely acknowledged. The compounds of graviola have shown promise as possible cancer-fighting agents and could be used to treat cancer. This review discusses bioactive metabolites present in graviola and their role in affecting the growth and death of different cancer cell types and the molecular mechanism of how it works to downregulate anti-apoptotic genes and the genes involved in pro-cancer metabolic pathways. Also, it reviews how simultaneously increasing the expression of genes promotes apoptosis and causes cancer cells to die so that the active phytochemicals found in graviola could be used as a promising anti-cancer agent.

**Abstract:**

The ongoing rise in the number of cancer cases raises concerns regarding the efficacy of the various treatment methods that are currently available. Consequently, patients are looking for alternatives to traditional cancer treatments such as surgery, chemotherapy, and radiotherapy as a replacement. Medicinal plants are universally acknowledged as the cornerstone of preventative medicine and therapeutic practices. *Annona muricata* is a member of the family *Annonaceae* and is familiar for its medicinal properties. *A. muricata* has been identified to have promising compounds that could potentially be utilized for the treatment of cancer. The most prevalent phytochemical components identified and isolated from this plant are alkaloids, phenols, and acetogenins. This review focuses on the role of *A. muricata* extract against various types of cancer, modulation of cellular proliferation and necrosis, and bioactive metabolites responsible for various pharmacological activities along with their ethnomedicinal uses. Additionally, this review highlights the molecular mechanism of the role of *A. muricata* extract in downregulating anti*-*apoptotic and several genes involved in the pro-cancer metabolic pathways and decreasing the expression of proteins involved in cell invasion and metastasis while upregulating proapoptotic genes and genes involved in the destruction of cancer cells. Therefore, the active phytochemicals identified in *A. muricata* have the potential to be employed as a promising anti-cancer agent.

## 1. Introduction

Cancer is the world’s most prevalent cause of death, accounting for roughly 10 million fatalities in 2020 [1] and approximately 400,000 children are diagnosed with cancer annually [2]. As reported by the International Agency for Research on Cancer (IARC), there were 17.0 million new cases of cancer in 2018. By 2040, population growth and aging are expected to increase the global cancer burden to 27.5 million new cases and 16.3 million deaths from cancer [3]. Despite these challenges, the estimated future cancer burden is likely to be much higher due to the acceptance of lifestyles linked to cancer risk. Lung, liver, stomach, colorectal, prostate, breast, and cervical/uterine cancer are probably the most frequently diagnosed cancers recorded in economically developing countries. Medical breakthroughs to treat and prevent this disease are always in demand [4,5,6], and attention toward plant-derived compounds reduces the risk of side effects compared to current chemotherapy treatments. Drug resistance, and notably multidrug resistance (MDR), whose ultimate consequence is apoptosis resistance, has long been regarded as the most significant barrier in cancer therapy. Natural products with multiple potential targets may aid in the restoration and maintenance of signaling network resilience. Consequently, natural products may be utilized to prevent or treat drug resistance in cancer treatment by boosting the intracellular concentrations of chemotherapeutic drugs by controlling MDR or activating alternative nonapoptotic cell death pathways, such as necroptosis, autophagy, and oncosis [7]. Due to their demonstrated safety, low cost, and oral bioavailability, dietary phytochemicals have several advantages over synthetic pharmaceuticals [8]. Plants have been used to treat various diseases since antiquity [9]. According to the World Health Organization (WHO), approximately 80% of the global population relies on traditional medicines, plant extracts, or plant-based substances for primary healthcare [10]. From the 1940s until the end of 2014, natural compounds or modified natural products accounted for 49% of the 175 small molecules authorized for cancer treatment [11,12]. According to the most recent information, of the 1881 approved medicinal entities over nearly four decades from 1981 to 2019, over 23% were derived from natural sources, i.e., unaltered natural products (N; 3.8%), botanical drugs (NB; 0.8%), or natural product derivatives (ND; 18.8%) [13]. An estimated 25% of the 247 newly approved anti-cancer drugs were derived from natural products [13].

Herbal medicine is a global icon of alternative medicines in most significant fields. Medical plant research is very much needed to promote the appropriate use of herbal medicine and to assess its potential to be developed as new drugs [14]. Plants with medicinal properties have been utilized to cure various ailments since before the dawn of history. Several references to medicinal plants and their disease-preventive properties are documented in ancient texts [15,16,17]. The key features were the chemical compounds of medicinal plants that can exert a physiological effect on the human system [18]. The secondary metabolites and bioactive compounds present in various medicinal plants have been studied and found to have anti-cancer effects [19]. Most of these natural substances exert their anticancer characteristics to restrict the initiation, development, and progression of cancer by influencing cellular proliferation, differentiation, apoptosis, angiogenesis, and metastasis [20]. *Annona muricata* (also known as graviola) extracts are among a plethora of botanical compounds that have demonstrated potential therapeutic efficacy and all of this plant’s aerial parts are utilized as natural medicines [21,22]. There have been reports of widespread use of *A. muricata* by people with cancer in numerous places throughout the globe [22]. In a Jamaican study on the opinions of cancer patients regarding using medicinal plants for self-medication, 60% of participants disclosed using *A. muricata* for the treatment of various malignancies, especially breast and prostate cancer [23]. Similarly, 80.9% of patients with breast, prostate, or colorectal cancer have been reported to use *A. muricata* in specialty oncology clinics in Trinidad [24]. Furthermore, a cross-sectional study suggests that 14% of Peruvian patients with liver cancer have used *A. muricata* to treat their cancer-related symptoms [25]. Similar claims of prostate cancer chemopreventive polyphenols in Nigerian foods containing *A. muricata* have been reported [26]. While the use of *A. muricata* leaf for cancer therapy has been reported in the Philippines [27] and Peru [28], it was reported to be utilized for gastric cancer therapy in Mexico [29].

The following sections discuss the research that has linked to *A. muricata*-derived compounds with anticancer effects, such as inhibition of proliferation, cytotoxicity, necrosis, and induction of apoptosis in several cancer cell lines. This article aims to overview *A. muricata* and its derivatives against various types of cancer, including pancreatic cancer, lung carcinoma, prostate carcinoma, breast cancer, colon carcinoma, head and neck squamous cell carcinoma, hematological malignancies, liver cancer, and cervical cancer.

## 2. Botanical Description and Distribution

The Annonaceae family includes about 130 genera and 2300 species, including *A. muricata* L., also known as soursop, graviola, guanabana, pawpaw, and sirsak [30,31]. *A. muricata* is native to the warmest tropical areas of South and North America, but now it has spread across the world’s tropical and subtropical countries, including India, Malaysia, Nigeria, Australia, and Africa [32]. Evergreen, terrestrial tree *A. muricata* grows to a height of 5 to 8 m with a broad, glossy, dark green, open, and round canopy. Individual yellow flowers on woody stalks are larger on this tree. The edible fruits of the tree are large, oval, or heart-shaped, green in color with more than 4 kg weight, with a diameter of 15 to 20 cm. The white juicy fibrous segments that make up the fruit pulp form an elongated receptacle. Fruit may have 5–200 seeds [33]. The skin is reticulated and has short spines, making it look leathery. It has a creamy, granular inner surface that easily separates from the soft pithy base [28].

## 3. Bioactive Metabolites Responsible for Various Pharmacological Activities in *A. muricata*

**Figure 1:**
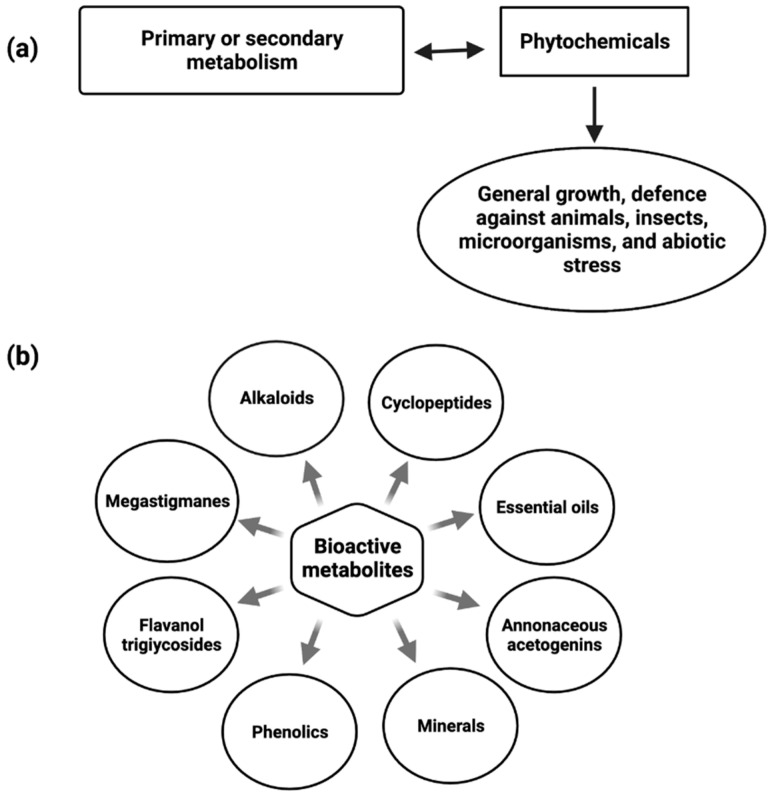
Diagram depicting the role of Phytochemicals (**a**) and bioactive metabolites present in *Annona muricata* (**b**).

Phytochemicals are constitutive metabolites produced by the primary or secondary metabolism of various parts of plants and have important plant functions (Figure 1). Plant growth and metabolism are also influenced by primary and secondary metabolites [34].

Alkaloids [35], megastigmanes, flavonol triglycosides [36], phenolics, cyclopeptides, and essential oils have all been found in various parts of the *A. muricata* (Figure 1). Despite this, Annona species, including *A. muricata*, are a good source of reported acetogenin (AGE) compounds. The presence of multiple major minerals in the *A. muricata* fruit, such as potassium, calcium, sodium, copper, iron, and magnesium, indicates that daily consumption of the *A. muricata* fruit will aid in the supply of vital nutrients and components to the human body [37].

### 3.1. Alkaloids

The alkaloids from *A. muricata* root are coclaurine, reticuline, argentinine, atherosperminine, and xylopine. Alkaloids were tested against HL-60 (human leukemia), A549 (human lung adenocarcinoma), and HepG2 (human liver carcinoma) cell lines; xylopine had the best cytotoxic activity (with IC50 of 20 to 80 µM) [38]. The alkaloids identified in the *A. muricata* peel were nornuciferin, assimilobin, anonaine, and isolaureline [39]. Xylopine and isocoreximine showed significant anti-cancer activity against A549 (human lung adenocarcinoma) and K-562 (human myelogenous leukemia) cell lines [38].

### 3.2. Phenolic Compounds

The ethyl acetate fraction of *A. muricata* leaf extract yielded kaempferol-3-O-glucoside and 1-(4-Hydroxyphenyl)-3-Phenylpropan-1-one, which has in vitro antioxidant activities [40]. Isolated polyphenols from *A. muricata* were kaempferol, and its isomers, procyanidins, catechin, and quercetin has cytotoxic activity against the HeLa (Henrietta Lacks) cancer line and 3T3 fibroblast cells [41]. The ethyl acetate and n-butanol fractions of *A. muricata* had increased total phenolic content and antioxidant capacity, as well as inhibitory activity against pancreatic lipase, α-amylase, and α-glucosidase [42].

### 3.3. Other Compounds

Other compounds found in *A. muricata* include vitamins, carotenoids, amides, and cyclopeptides. Vitamins and carotenoids were found in the fruit’s leaves, seeds, and pulp [43,44]. The seeds contain amide N-p-coumaroyl tyramine and cyclopeptides [45], which have anti-inflammatory and anti-tumor effects. The fruit pulp of *A. muricata* contains 37 volatile chemicals, the bulk of which are aromatic and aliphatic esters [46]. There were also 80 essential oils mentioned on the leaf, mostly sesquiterpene derivatives [47,48,49]. The analysis of *A. muricata* volatiles is promising due to their bioactivity [50].

### 3.4. Annonaceous Acetogenins

Acetogenins (AGEs) are a type of bioactive substance found in the *A. muricata* plant and some of the acetogenins are Annomuricin A, B, and C, Muricatocin C, cis*-G*oniothalamicin, Muricatacin, Arianacin, Annonacin-10-one, cis-Annonacin, and Javoricin [51]. The isolated compound from the *A. muricata* revealed the presence of hydroxyl functional groups and α, β-unsaturated γ-lactone and elucidated as 15-acetyl guanacone, possess radical scavenging activity against DPPH radical, ABTS radical [52]. Annonacin, isolated acetogenin, possesses cytotoxic activity against the Raji (human B lymphoblastoid) cell line, and the IC50 value was found to be 2.89 ± 1.3 (µM) and antioxidant activity against DPPH, ABTS radicals, and FRAP [53]. Treatment of MCF-7 (human breast cancer) cells with 10 μg/mL of Annonacin indicated marked genotoxic activity [54]. AA005 is an Annonaceous acetogenin mimic that inhibited tumor growth by promoting nuclear translocation of apoptosis-inducing factor (AIF) and inducing AIF-dependent cell death in vivo human colon cancer cell lines. In vivo SW620 (human colorectal cancer) and in vitro RKO (poorly differentiated colon carcinoma) cell lines revealed that treatment with AA005 showed down-regulation in Bcl 2 and Mcl-1 [55]. In silico research on acetogenins reverse ABCB1-mediated multidrug resistance in colon cancer: Annohexocin, Annomuricin, Annomuricin B, Annomuricin E, Annonacin A, Annonacin, Annonacin-10-one, Annonacinone, Annopentocin, Annopentocin B, Annopentocin C, Cis-corossolone, Corossolone SL, Epomuricenin A, Epomuricenin B, Gigantetrocin A, Gigantetronenin, Goniothalamicin, Muricapentocin, Muricatocin A, Muricatocin B, Muricoreacin, Solamin revealed that Annonacin, Annohexocin and Annomuricin showed the highest docking scores [56].

**Figure 2:**
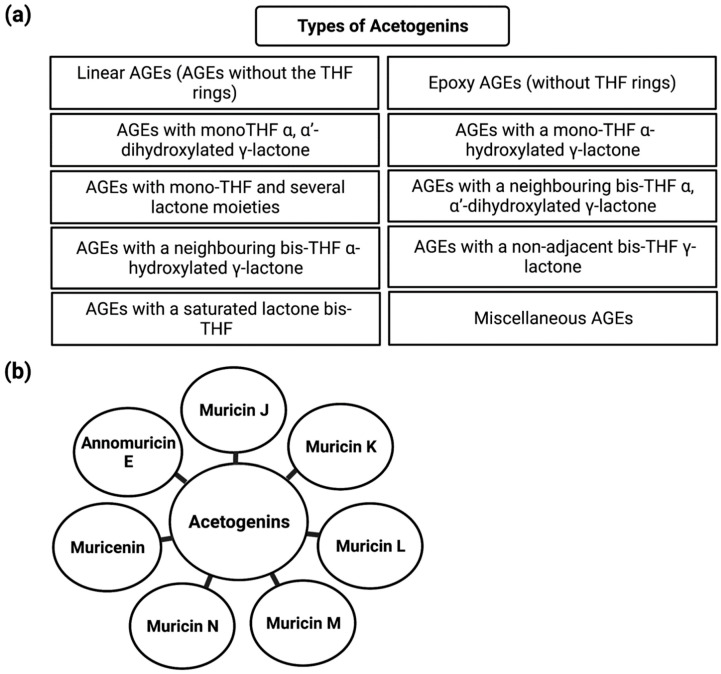
Schematic representation of the types of acetogenins (**a**) and cell cycle machinery regulated by acetogenins (**b**).

**Figure 3:**
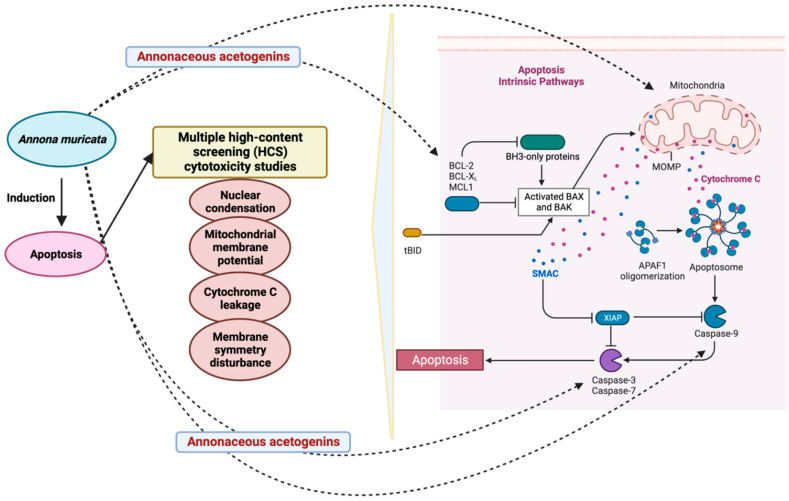
Role of *A. muricata* in apoptosis. The figure was created with BioRender.com.

More than 500 Acetogenins have been recorded from various parts of the plant, and these acetogenins specifically target cancer cells without harming normal cells [57]. Following that, these metabolites were divided into several categories based on hydroxyl groups, tetrahydrofuran, terminal γ-lactone ring, and aliphatic chain substituents. AGEs can be classified into ten different categories based on these features (Figure 2). Acetogenins are able to inhibit mitochondrial ATP formation. This molecular mechanism has been demonstrated to be efficient against cancerous cells that create more ATP than normal cells, inhibiting cancer cell growth [58]. The proteolytic cleavage of poly (ADP-ribose) polymerase-1 (PARP-1), a nuclear enzyme involved in DNA integrity, DNA repair, and transcriptional control, is a signature of apoptosis. PARP-1 is cleaved by caspases, in particular caspase-3 and -7 [59]. PARP-1 cleavage has been proposed to serve as a molecular switch between apoptotic and necrotic forms of death receptor-induced cell death, as ATP is necessary for apoptosis [60]. Acetogenin’s mechanism for inducing the apoptotic process involves blocking ATP production by inhibiting the NADH: ubiquinone oxidoreductase (Complex I) in the electron transport system in the mitochondria of cancer cells. This results in the cancer cells losing their supply of energy and becoming weaker, ultimately leading to cell death [61,62]. Including the methyl group on the γ-lactone moiety makes natural acetogenins potent mitochondrial complex I inhibitors [63]. Bax and Bcl-2 protein expression involvement is critical in determining tumor responsiveness to a specific anticancer treatment [64]. Changes in mitochondrial membrane permeability and cytochrome c release can be attributed to the Bcl-2 protein (Figure 3). Once the mitochondrial release of cytochrome c is no longer blocked due to a decrease in Bcl-2 expression, apoptosis is induced, and the caspase pathways are activated to generate caspase-3 as apoptotic executors [65]. Apoptosis can be induced by acetogenin by decreasing the expression of antiapoptotic proteins such as Bcl-2 and Bcl-xL, thereby allowing the expression of Bax and Bad pro-apoptotic proteins to increase [66] and by enhancing the expression of caspase 3/7 and caspase 9 (Figure 3) [66,67].

## 4. Ethnomedicinal Uses

Due to their therapeutic potential, the Annonaceae family has been widely investigated in recent years. The medicinal uses of the Annonaceae family have been recorded for a long time, and this species has gained attention in recent years due to its bioactivity and traditional uses [50]. Medicinal herbs are widely recognized as the foundation for human life and health care. Chronic degenerative diseases have attained widespread levels and are now regarded as serious health problems; as a result, treatment of these diseases is given clinical priority [68]. *A. muricata* has been proposed as an insecticide [69] and a parasiticide [70] in ethnobotanical studies. Fever [70], sedative [71], respiratory disease [70], malaria [72,73], gastrointestinal disorders, liver, heart, and kidney disorders [74,75,76] have all been treated with fruit juice and leaf/branch infusions. In recent years, this has been widely used for hypoglycemic [42], hypotensive [70], and cancer treatments [77,78] (Table 1).

The leaves [96,97,98,99,100], pericarp [36,101], fruits [34,102], seeds [36,103], and roots [35] of *A. muricata* have ethnomedical uses. Roots of *A. muricata* have been employed in herbal therapy, whereas stem barks, stems, seeds, and leaves are the most common constituents in traditional medicinal decoctions [104,105,106]. In *A. muricata* extracts, Coria-Téllez et al. [70] found 212 bioactive chemicals. Traditional medicine uses different plant parts to cure a range of diseases and ailments, including inflammation [77], rheumatism, diabetes [42], hypertension [107], and parasite infestation [82]. The fruit is often used to treat arthritis and fever, whereas the seed is used to treat worms. Parasitic diseases can be handled with seeds and fruits as well. The leaves are also used as a traditional medicine for treating collapses [108], hypoglycemia, inflammation, and spasm relief treatment [30]. Furthermore, the plant’s leaf has been nicknamed “the cancer killer” and is often used in conventional cancer care medicine, as the name suggests [91,109]. It is well known that the plant was widely used as a source of chemically active metabolites due to its various curative properties [109].

## 5. Role of *Annona muricata* against Various Types of Cancer

### 5.1. Pancreatic Cancer

Pancreatic cancer, the deadliest malignancy in the world and the 4th largest source of malignancy fatalities, seems to have a 5-year survival rate of only 8% [110]. Due to a lack of early clinical symptoms, there is a high mortality rate in late diagnosed patients. Late diagnosis, resistance to available chemotherapy treatments, and cancer’s high aggressive behavior have encouraged new early detection markers, as well as research and evolution of chemo-preventive and chemotherapy agents. Even though several plant chemicals have been studied for pancreatic cancer treatment, none have yet been scientifically proven [111].

**Figure 4:**
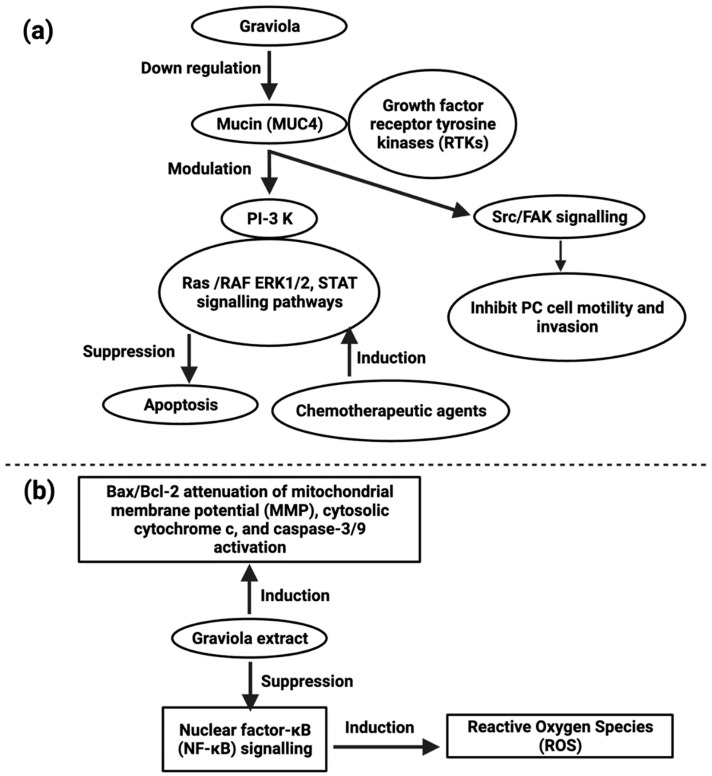
Representation of the role of MUC4 in pancreatic cancer (**a**) and action of graviola extract against lung cancer (**b**).

*A. muricata* capsules containing leaf and stem powder have anti-proliferative and antitumor effects in pancreatic cancer cell lines (IC50 values were 200 µg/mL in FG/COLO357 and 73 µg/mL in CD18/HPAF), and in subcutaneous xenografts, these activities included inducing cell cycle arrest with apoptosis. The migratory capacity of pancreatic cells has been similarly diminished upon treatment with the extract at a concentration of 100 µg/mL by a transwell assay [112]. Similar anti-proliferative effects have been identified for the hexane fraction of *A. muricata* leaves against pancreatic cancer cell line, Capan-1 (IC25 values were 7.8–8 μg/mL), which is rich in flavonoids [113]. *A. muricata* also inhibited the motility and invasion of PC cells by downregulating the mucin MUC4 (Figure 4) [114]. Despite MUC4′s enormous pathological importance in various cancers, *A. muricata*’s high therapeutic suitability for various tumors, especially PC, is indicated by MUC4 down-regulation. *A. muricata*, in addition to downregulating MUC4, has been shown to cause cell death by modifying glucose metabolism and inducing metabolic catastrophe [112,115].

Recently, anticancer strategies targeting cancer cells’ metabolism have received considerable attention. Tumor cells need inexorably more energy to proliferate, which is obtained from glycolysis. Recent research suggests that KRAS controls glycolysis by upregulation of the expression of GLUT1 in conjunction with HK1, HK2, PFKl, and LDHA [116]. When inhibiting KRAS, decreased cellular metabolism has been associated with anticancer effects [117].

To achieve this anti-proliferative effect, it may be necessary to modify glucose levels by lowering glucose uptake or altering GLUT expression. The metabolic catastrophe caused by *A. muricata*, which was triggered by the downregulation of HIF-1, GLUT1, GLUT4, HK2, and LDHA in PC (prostate cancer) cells, was associated with decreased glucose absorption and ATP generation [112]. Bullatacin-type of acetogenin promotes cytotoxic effects by regulating metabolic processes and suppressing the mitochondrial complex I proton pumping mechanism, which inhibits ATP generation and NADH oxidation [115]. The components in the *A. muricata* extract suppress signaling pathways involved in PC cell formation, proliferation, and metabolism. The cytotoxicity of *A. muricata* in cancer cells has prompted scientists to investigate deeper into the molecular mechanisms behind these results (Table 2).

According to Torres [112], the activation of ERK and PI3K pathways plays a critical role in pancreatic cancer cell survival, and inhibiting these pathways contributes to pancreatic cell growth inhibitors. In a similar study, pancreatic cells treated with *A. muricata* extracts reduced the ERK and Akt pathways activation. As a result, the reduced viability of pancreatic cells administered with plant extract [112] is consistent with the inhibition of these pathways. *A. muricata* was also found to be inhibited by metastasis. Torres’ research on pancreatic cells showed that after treatment with an *A. muricata* extract, pancreatic cancer cells’ migratory capacity was reduced, as measured by a transwell assay, implying that herbal extract reduces pancreatic cancer cell motility. The cortical actin and microtubule network composition influences cancer cell motility and migration. Furthermore, cellular ATP depletion has been linked to cytoskeleton actin reorganization and a suspension of microtubule dynamics, which are thought to cause mitotic arrest. *A. muricata* extracts disrupt the cortical actin network, stopping cancer cells from moving around [112].

### 5.2. Lung Carcinoma

Lung cancer is the most common cancer-related death worldwide [110]. Because of chemotherapeutic tolerance, many patients with lung cancer succumb to the disease. In vitro studies of A549 (human lung adenocarcinoma) cell lines revealed that *A. muricata* leaf extract possesses cytotoxic activity and the IC50 values for hexane, ethyl acetate, and methanol extracts were 21.05 ± 0.42 µg/mL, 5.09 ± 0.4 µg/mL and ≥ 100 µg/mL respectively, caused cell cycle arrest at the G_0_/G_1_ phase and apoptosis (Figure 4) [121]. cis-Annonacin-A-one and trans-Annonacin-A-one, cis- gigantetrocinone and trans-gigantetrocinone, cis-isoAnnonacin and trans-isoAnnonacin and squamolone isolated acetogenins possess cytotoxic activity against A549 cell lines, and the ED50 values were 3.39 × 10^−2^, 9.74 × 10^−3^, 4.42 × 10^−5^, ≥ 10, 1.48 × 10^−3^, respectively [122].

*A. muricata* leaf ethyl acetate extract (AMEAE) possesses a cytotoxic effect on the A549 cell line, and the IC_50_ value was 5.09 ±  0.41 μg/mL after 72 h of treatment, inducing apoptosis. This was proved by multiple high-content screening cytotoxicity studies. The results showed that A549 cells treated with the *A. muricata* extract inhibited growth potential, and their apoptosis pathway was upregulated [121]. Graviola extract inhibits nuclear factor-κB (NF-κB) signaling, increases ROS production, and enhances the Bax/Bcl-2 ratio–mediated inhibition of mitochondrial membrane potential, activation of cytosolic cytochrome c, and caspase-3/9 as reported in A549 cell line [121].

### 5.3. Prostate Carcinoma

Prostate carcinoma is the highest source of cancer-related fatalities in Western developed countries [110], with over 1,64,690 records and 29,430 deaths in 2018. Though rapid innovations in early identification and novel therapy techniques can significantly boost these patients’ lives, a significant proportion of them acquire aggressive and refractory tumors with a bad prognosis. Several bioactive compounds have been tried as adjuvants to existing therapy for treating and preventing hormone-refractory pancreatic cancer, but no clinical success has been found [123].

**Figure 5:**
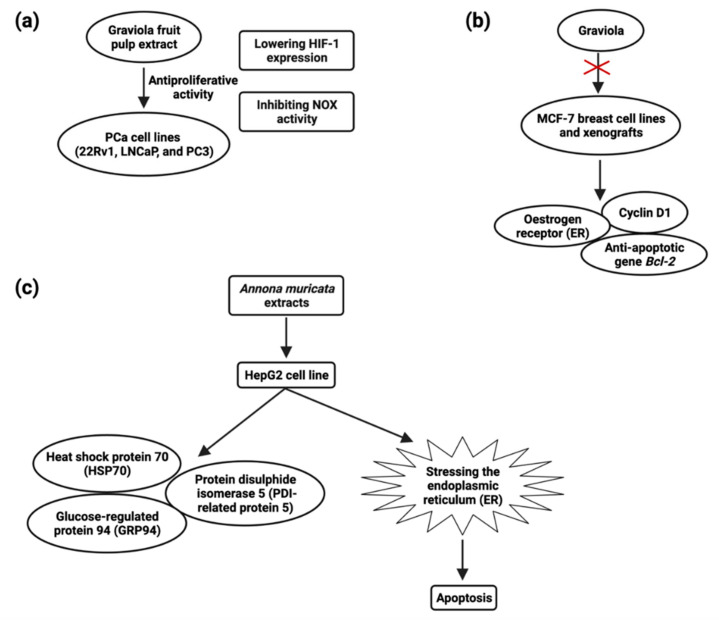
Schematic presentation of the anti-proliferative activity of graviola fruit extract against prostate cancer cell lines (**a**), the role of graviola against breast cancer cell line MCF-7 (**b**), and the molecular mechanism of *A. muricata* extract-treated HepG2 cell (**c**).

MTT and colony formation assays revealed that *A. muricata* fruit pulp extract has potent antiproliferative activity in prostate cancer (PCa) cell lines 22Rv1, LNCaP, and PC-3 at a concentration of 1–5 μg/mL [124]. They also discovered that fruit extract had antiproliferative effects, which were mediated by lowering HIF-1 expression and inhibiting NOX activity (Figure 5) [125]. Muricin J, muricin K, and muricin L induced antiproliferative and apoptotic effects on PC-3 cells at 20 µg/mL [126]. Muricin M, muricin N, and muricenin obtained from the *A. muricata* fruit bioactive ethanolic extract were shown to have antitumor effects at a concentration of 20 µg/mL [124].

*A. muricata* leaf extract, the fraction with enriched flavonoids and acetogenins, possesses antiproliferative activity. The IC50 values were 63 µg/mL, 57 µg/mL, and 87 µg/mL against the prostate cancer cell line, PC-3. This study has proven the relevance of employing whole-leaf extracts to achieve maximum inhibitor potency in cancer treatment [127] (Table 3).

Aqueous leaf extract possesses antiproliferative activity against BPH-1 (human benign prostatic hyperplasia) cells; cell viability decreased from 100% to 47% as the dose increased from 0 to 1.5 mg/mL, and aqueous extracts of the leaf were found to minimize prostate size, which may be because of apoptosis [131]. According to the available evidence, antiproliferative activity has been demonstrated by muricins J, K, L (20 µg/mL) [126] and muricins M, and N along with muricenin (20 µg/mL) [124]. Regarding antiproliferative activity of annomuricin E, its IC50 value on HT-29 cells was found to be 5.72 ± 0.41 μg/mL (12 h treatment), 3.49 ± 0.22 μg/mL (24 h treatment) and 1.62 ± 0.24 μg/mL (48 h treatment) (Figure 2) [67]. Solvents including hexane (*A. muricata* fruit extract, 20 µg/mL), ethyl acetate extract [37], methanol leaves extract IC50 value against HEP-2 cell line (54.92 ± 1.44 μg/mL) [132], ethanol extract, and water extract [133] have antiproliferative impacts. Muricin J, muricin K or muricin L, muricin M, and muricin N, as well as muricenin, showed antiproliferative effects against PC-3 cancer cells [124,126].

### 5.4. Breast Cancer

The most prevalent malignancy among women worldwide is breast cancer [110]. Early-stage breast cancer may be treated, but advanced breast cancer has no treatment options. New chemopreventive and chemotherapeutic drugs are desperately needed in order to impede the formation of tumors and reduce associated morbidity. Although certain natural chemicals have been examined in vitro and shown to be safer and less hazardous than synthetic compounds [134], these natural compounds’ low clinical efficacy has hampered their translational use. Recent studies have demonstrated *A. muricata*’s strong antiproliferative and antitumor ability. Exposure of cancer cells to *A. muricata* leaf extract and ethyl acetate fraction resulted in morphological alterations indicative of apoptosis, a process characterized by the rupture and loss of the membrane and nucleus of cells. Reduced Bcl-2 and PARP-1 and increased caspase-9 and caspase-3 expression are responsible for the cytotoxic activity observed in MCF7 cells [62]. Treatment of Annonacin (0.5–1 µM) induced MCF breast cancer cell line death at 48 h and treatment of Annonacin (0.1 µM) in xenografts tumor decreases the expression of ER, cyclin D1, and Bcl2 (Figure 5) [135].

*A. muricata* fruit extract inhibited MDA-MB-468 cells (IC50 = 4.8 µg/mL) and significantly downregulated EGFR mRNA expression, cell cycle arrest, and apoptosis but not in MCF-10A cell lines. In the xenograft mouse model, 5-week dietary treatment with fruit extract (200 mg/kg diet) decreased expression of EGFR, p-EGFR, and p-ERK in MDA-MB-468 tumors by 56–32.5% [136]. MDR is the predominant source by which tumor cells gain therapeutic resistance, leading to therapy failure and tumor progression. By depleting ATP content, ACG bullatacin (1 µg/mL) is cytotoxic to the MCF-7/ADR cell line of multidrug resistant breast cancer [137]. ACGs from *A. muricata* offer a particular benefit against MDR breast tumors, whereas ACGs extracted from Annona Squamosa seeds alter mitogen-activated protein kinase (MAPK) signaling and triggered apoptosis in MCF-7/ADR cells. Annosquacin B (AB), dramatically reduced cell viability on MCF-7/ADR (IC50value-14.69 µM), induced apoptosis followed by elevated levels of caspase-3, caspase-9, Bax/Bcl-2, p-p38 MAPK and lowered p-JNK [138] (Table 4).

### 5.5. Colon Carcinoma

CRC (colorectal carcinoma) is the third most common cause of cancer-related fatalities [110]. The biggest problems for CRC patients are therapeutic resistance and toxicity against current medications, which are connected to a poor prognosis [145].

**Figure 6:**
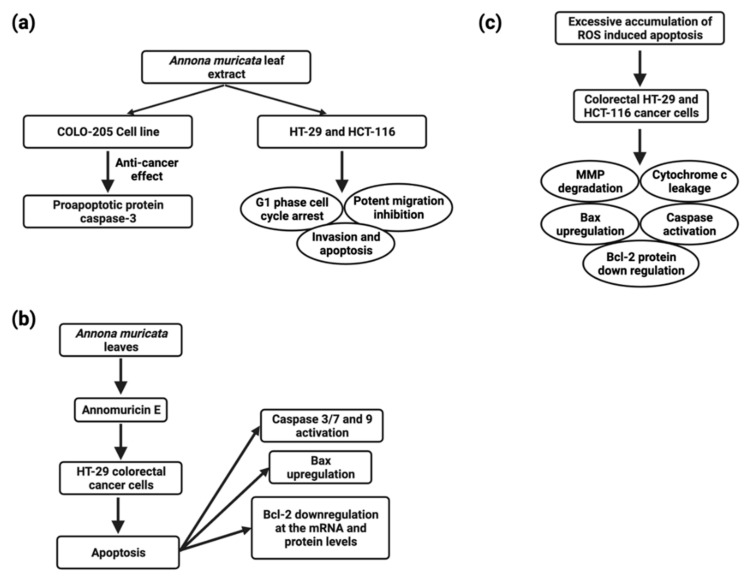
Schematic representation of molecular mechanisms of *Annona muricata* extracts against CRC (**a**), apoptosis induction in Annomuricin E treated HT-29 colorectal cancer cells (**b**), and apoptosis induction and mechanism of action in colorectal cancer-treated cell lines (**c**).

*A. muricata* and other natural products have proven to be effective in preventing and treating CRC. The molecular mechanisms of various *A. muricata* extracts against CRC have recently been elucidated. In the COLO-205 (human colon adenocarcinoma) cell line, the *A. muricata* leaf extract (1422 ng/mL) also showed anticancer properties by increasing the proapoptotic protein caspase-3 [146] (Figure 6). The ethyl acetate leaf extract of *A. muricata* has a substantial cytotoxic effect on HCT-116 (human colorectal carcinoma) and HT-29 (human colorectal adenocarcinoma) cell lines (Figure 6), and IC50 values were 11.43 ± 1.87 µg/mL and 8.98 ± 1.24 µg/mL, respectively, followed by cell cycle arrest, apoptosis, mitochondrial membrane depolarization, cytochrome c leakage and activation of the initiator and executioner caspases, up-regulation of Bax and down-regulation of Bcl-2 proteins, halted the migration and invasion of HT-29 and HCT-116 cells [109].

Phytochemical constituents of *A. muricata*, such as Annomuricin A (ED50 value of >1.0 µg/mL), Annomuricin B (ED50 value of 4.35 × 10^−1^ µg/mL), Annomuricin C (ED50 value of 1.54 µg/mL), Annomuricin E (ED50 value of 6.68 × 10^−2^ µg/mL), and muricapentocin (ED50 value of 7.10 × 10^−^2 µg/mL), were also found to be cytotoxic to HT-29 cells [147,148,149].

ACG desacetyl uvaricin (IC50 value of 14 nM) has also been shown to cause DNA damage by inactivating the MAPK pathway and producing superoxide, resulting in the growth inhibition of SW480 cells [150] (Figure 6). Annomuricin E derived from *A. muricata* leaves induced apoptosis in HT-29 cell line via caspase activation 3/7 and 9, Bax upregulation, and Bcl-2 down-regulation at the mRNA and protein levels (Figure 3).

Annomuricin E halted HT-29 cell proliferation, HCT-116 with an IC50 value of 1.62 ± 0.24 μg/mL, 32.51 ± 1.18 μg/mL after 48 h, followed by G1 cell cycle arrest and excessive accumulation of ROS-induced apoptosis in colorectal HT-29 and HCT-116 cancer cells, which was accompanied by MMP degradation, cytochrome c leakage, initiator and executor caspase activation, Bax upregulation, and Bcl-2 protein down-regulation (Figure 6). In rat colorectal carcinogenesis caused by azoxymethane, the ethyl acetate leaf extract reduced the inhibition of aberrant crypt foci by 72.5 % [67] (Table 5).

### 5.6. Head and Neck Cancers

**Figure 7:**
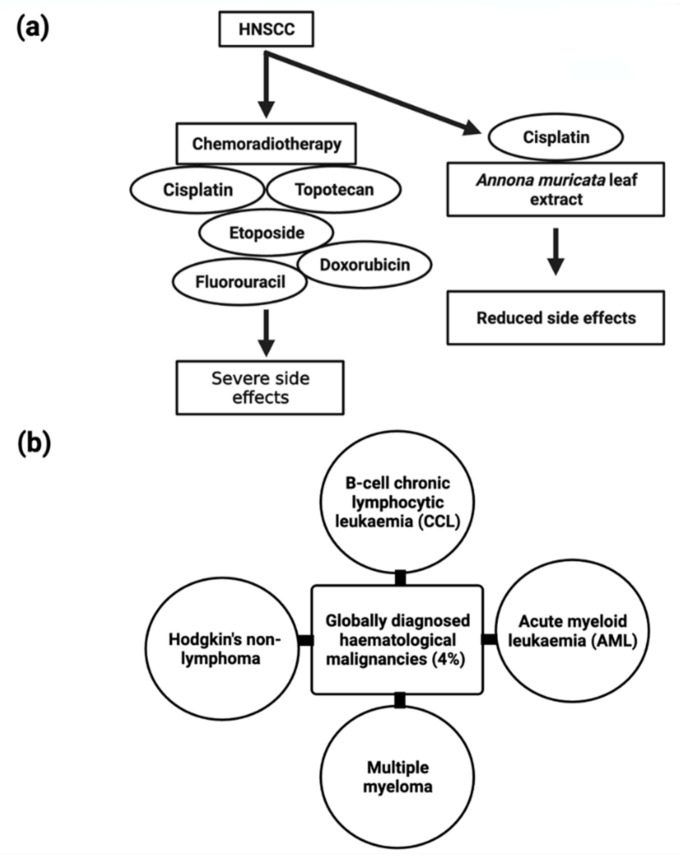
Schematic representation of (**a**) comparison of chemoradiotherapy treatment and combination of Cisplatin and *Annona muricata* extract treatment for HNSCC and their side effects and (**b**) globally diagnosed types of hematological malignancies.

The majority of head and neck malignancies arise from the mucosal epithelium of the oral cavity, pharynx, and larynx, and are generally referred to as head and neck squamous cell carcinoma (HNSCC) [151] and are the sixth highest prevalent cancer worldwide. Several anticancer drugs, such as etoposide, cisplatin, topotecan, doxorubicin, and fluorouracil (Figure 7), have been linked to adverse side effects that severely limit their utility. Through current advancements in the understanding and treatment of the disease, patients with HNSCC have a poor prognosis due to resistance to available chemoradiotherapy. As a result, there is a critical need to investigate less toxic anticancer compounds.

Aqueous *A. muricata* leaf extract possesses antiproliferative activity against the tongue squamous cell carcinoma patient-derived cell line SCC-25 (12.42 µg/mL) and induces G2/M cell cycle arrest [133]. With radiation therapy or in combination with other chemotherapeutic medicines, cisplatin’s efficacy against HNSCC increases dramatically, suggesting that cisplatin mixed with *A. muricata* could have significant anticancer effects on HNSCC [152]. Radiation is well-known to cause DNA damage, which initiates apoptosis, which is controlled by the proteins p53, Bcl2, and Bax (Figure 3). For radiation-induced apoptosis, a high amount of wild-type p53 is necessary. Therefore, the p53 status may be a critical factor in determining the radiosensitivity of tumor cells. The radiosensitivity of patients with head and neck cancer is determined by the ratio of p53, Bcl2, and Bax protein levels. Patients with cancer who had elevated Bcl2 levels were radioresistant. Overexpression of Bax and c-myc may increase head and neck cancer patients’ radiosensitivity [153]. Bax and c-myc overexpression may guarantee radiosensitivity in patients with head and neck cancer [153]. Therefore, the combination of *A. muricata* extracts with cisplatin would be more successful due to its involvement in inducing apoptosis.

The ability of a leaf fraction from *A. muricata* to induce caspase 3 expression in cells cultivated from WHO stage III nasopharyngeal carcinoma biopsy tissue was studied. Compared to the control group, the treatment group had increased caspase 3 expression. The maximum expression was attained 24 h after treatment. Caspase 3 expression increased with increasing dosages of *A. muricata* leaf fraction, most notably at 125 µg/mL and 250 µg/mL [154]. Activating caspase 3/7 and caspase 9 expression is a key step in inducing apoptosis, which is caused by the *A. muricata* leaf fraction (Acetogenin) [67].

### 5.7. Hematological Malignancies

B-cell chronic lymphocytic leukemia, acute myeloid leukemia, multiple myeloma, and non-lymphoma Hodgkin’s make up about 4% of cancers diagnosed globally (Figure 7) [110]. Despite the disease’s biological characteristics and complexity, it poses particular clinical challenges. In clinical trials, phytocompounds and derivatives have been evaluated, but the majority of compounds have been unsuccessful due to inadequate effectiveness, resulting in resistance; therefore, it is imperative to develop novel natural chemical compounds with improved therapeutic capabilities. Though random screening is a high-priced and tedious process, focused research into commonly used medicinal plants can speed up the development of new anticancer drugs [155].

*A. muricata* twig, root, and leaf extract possess antiproliferative activity, and the IC50 values were found to be 49 ± 3.2 µg/mL, 9 ± 0.8 µg/mL, 14 ± 2.4 µg/mL, respectively, and apoptotic effects have been linked to cell cycle arrest and MMP loss in mechanical experiments [156]. Furthermore, ethanol and methanol *A. muricata* leaf extract-induced apoptosis in K562 (human myelogenous leukemia), CCRF-CEM (human T-leukemia), and CEM/ADR5000 (multidrug-resistant leukemia) cells have been reported [101]. In this study, it was found that *A. muricata* can be used to identify phenotypes of multidrug-resistant malignancy and is thus an outstanding method for the evolution of new therapeutic medicines for hematological malignancies.

Methanolic extracts from seeds, leaves, and pericarp have shown antiproliferative activity, and the IC50 values were found to be fruit pericarp 4.58 ± 0.25 µg/mL, leaves 0.57 ± 0.02 µg/mL, and seeds 0.36 ± 0.03 µg/mL against CCRF–CEM cells and induced apoptosis, whereas pericarp and leaf extracts induce apoptosis in leukemia cells CEM/ADR5000 [37]. An ethanolic leaf extract at a 50 µg/mL concentration significantly increased caspase3 activity to induce apoptosis in K562 leukemia cancer cells, as reported by the TUNEL assay [157] (Table 6).

### 5.8. Liver Cancer

The plant extracts have been shown to have a cytotoxic impact on hepatic cancer cells, hinting that they could be employed as a hepatic cancer treatment. The HepG2 cell line was shown to be inhibited in growth and viability after being incubated with an ethanol extract of *A. muricata*; after 24 and 48 h of treatment and LD50 values were 180 and 80 µg/mL, respectively, and apoptosis through inducing ROS pathway [159]. *A. muricata* leaf extracts possess antiproliferative activity, and the IC50 values were 150 μg/mL for both HepG2 and HCT116 cells, inducing apoptosis at a concentration of 120 μg/mL against HepG2 cells. HSP70, GRP94, and PDI-related protein 5 (Figure 5) were all found to be upregulated after HepG2 treatment [160] (Table 7).

### 5.9. Cervical Cancer

In HeLa cervical cancer cells, different solvent leaf extracts were able to induce antiproliferative activity (Table 8). As shown by the (3-[4,5-dimethylthiazol-2-yl]-2,5-diphenyltetrazole) (MTT) reduction method, *A. muricata* methanol leaf extract was able to inhibit the proliferation of the HEp-2 (laryngeal cancer) cell line [132]. However, later HEp-2 was confirmed to be a HeLa cell line that had been cross-contaminated [162].

## 6. Potential Role of *A. muricata* as a Modulator of Epithelial-Mesenchymal Transitions (EMTs)

**Figure 8:**
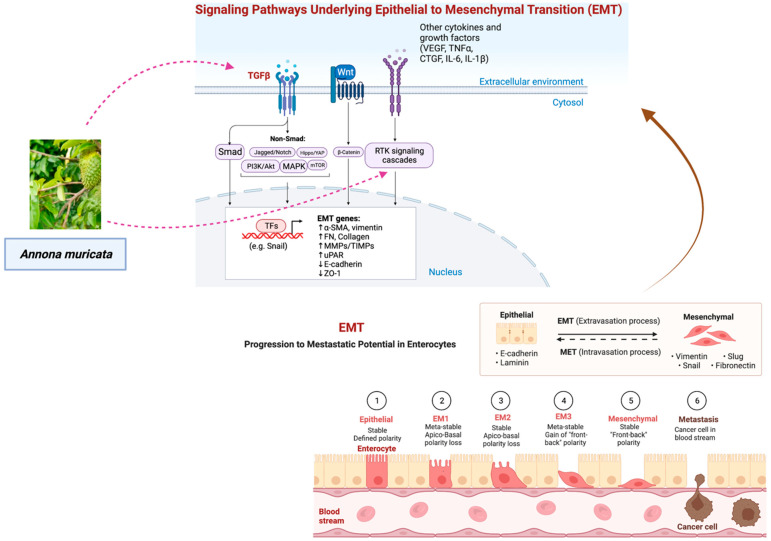
Regulation of epithelial-mesenchymal transition (EMT) by signaling pathways. The EMT process is activated by many signaling pathways involving extracellular cytokines and receptor tyrosine kinases (RTKs). TGFβ is the master regulator, activating not just the canonical Smad signaling pathway but also numerous other signaling pathways. It has been shown that the Wnt/-catenin signaling pathway is also crucial to EMT. Upon activation of EMT, the transcription factors (TFs) of the snail family and mesenchymal genes are upregulated, while the epithelial genes are downregulated [165,175]. The figure was created with BioRender.com.

Epithelial-mesenchymal transitions (EMTs), the acquisition of mesenchymal characteristics by epithelial cells, occur during certain biological processes (defined as subtypes 1 and 2) as well as cancer invasion and metastasis (the third subtype of EMT) [164]. EMT is activated by many signaling pathways involving extracellular cytokines and receptor tyrosine kinases (RTKs) [165]. Cancer metastasis begins with EMT, a complex process in which normally immobile epithelial cells undergo a morphological change into motile mesenchymal-appearing cells. This causes cancer cells to detach from the primary site by reducing their cell-to-cell junctions and allowing them to migrate to distant sites [164,166] (Figure 8). Epithelial marker E-cadherin, a junction protein mediating cell-cell contact, can be downregulated in EMT-phenotypic cells. In addition, these cells can stimulate transcription factors of EMT switch, such as Slug and Snail, and raise levels of mesenchymal markers including N-cadherin, β-catenin, and vimentin (Figure 8) [167]. However, EMT does not merely just facilitate cellular migration during metastasis, it also impacts resistance to apoptosis, particularly the detachment-induced apoptosis anoikis [168], blocks senescence, boosts survival, facilitates genomic instability, leads to cancer stem cell activity, increases drug resistance, modulates metabolism, and suppresses the immune system [169,170]. Cancer recurrence at the metastatic site is believed to need the reverse process, termed mesenchymal to epithelial transition (MET) (Figure 8), following invasion and propagation [171]. For the reversion of migratory mesenchymal neoplastic cells and the subsequent formation of macrometastases, microenvironmental cues are regarded as a crucial deterministic component. When MDA-MB-231 human breast cancer cells were cultivated with rat hepatocytes, re-expression of E-cadherin, suppression of SNAIL, and sequestration of β-catenin revealed evidence of MET in liver metastasis [171,172].

As EMT has been connected to the metastatic tendencies of cancer cells, natural products derived from *A. muricata* could be used to develop anti-metastasis therapeutics. By the induction of EMT-related transcription factors, it has been proven that the transforming growth factor beta (TGF-β)/Smads pathway is the most potent EMT inducer [173]. Previous research has shown that the EMT can be triggered by the transforming growth factor-beta 1 (TGF-β1), which in turn encourages the migration and invasion of lung adenocarcinoma cells. It may be possible to utilize an extract of the leaves of *A. muricata* to inhibit the activity of TGF-β1 and hence control subtype 3 EMT. Interestingly, the levels of transforming growth factor-beta 1 (TGF-1) in liver tissue were found to be reduced in an in vivo study that evaluated the effect of the ethanolic extract of *A. muricata* (250 mg/kg and 500 mg/kg) on thioacetamide (TAA)-induced liver cirrhosis in Sprague Dawley rats. Acute oral toxicity tests on rats showed no harmful symptoms or fatalities, confirming *A. muricata*’s safety [174].

While by serving as an immunomodulatory switch, members of the glycoprotein family, such as MUC1, can play either a pro- or anti-inflammatory role in various infection-induced malignancies [5,176], MUC4 plays an important role in the etiology of many different types of cancer, including pancreatic cancer (PC), ovarian cancers, and head and neck cancers (HNC) [114,152]. MUC4 suppresses apoptosis and induces resistance to several chemotherapeutic agents by modulating STAT and the PI-3K, Ras/RAF/extracellular signal-regulated kinase (ERK1/2) signaling pathways through physical interaction and stabilization of the ErbB family of growth factor RTKs (Figure 8) [114,165]. Graviola not only reduces MUC4 expression but also causes a metabolic catastrophe and cytotoxicity [112], thus reducing migration and invasion of pancreatic cancer cells [152].

## 7. Toxicological Studies

Extracts from *A. muricata* have been studied for their potential to treat cancer; however, there are currently no available data on their safety. AGEs are environmental neurotoxins responsible for neurodegenerative tauopathy, as suggested by a study in the Caribbean island of Guadeloupe, which found a correlation between AGE consumption and the prevalence of the neurodegenerative disease [177]. *A. muricata*’s primary acetogenin, annonacin, has been shown to kill striatal neurons in vitro and promote the redistribution of tau protein from the axon to the cell body [177]. Due to the fact that the content of potential toxins can vary based on the plant’s location, extraction method, plant part, and harvest time, the other toxicologic findings discussed above in the individual studies merit serious consideration, and future studies involving the use of graviola components must involve rigorous safety testing. Seven different acetogenins were tested for neurotoxicity using rat striatal neuronal cells, mesencephalic dopaminergic neurons, and laboratory rats, and the results showed that *A. muricata*’s most prevalent acetogenin (annonacin) and alkaloid (reticuline) were neurotoxic [37,67]. However, studies on the neurotoxicity of annonacin suggest that prolonged exposure to this molecule is required in order to evaluate the effect in mouse models, and pharmacokinetic studies also predicted that this compound had a low degree of bioavailability [50]. In 2010, however, researchers agreed that consuming Annonaceae species did not have a causal effect on the development of atypical parkinsonism [50].

## 8. In Vivo Studies

Graviola extract treatment was reported to offer antioxidant defense with significant chemopreventive and chemotherapeutic efficacy against 7,12-dimethylbenza(α)anthracene (DMBA)-induced breast carcinogenesis in Swiss albino mice [178]. None of the tumors in the cancerous control group displayed necrosis, as determined by histological examination. Significant regions of necrosis were seen in tumors removed from rats treated with graviola extract at doses of 100 and 200 mg/kg body weight, compared to the 50 mg/kg group [178]. Breast cancer prognostic markers such as survivin, livin, osteopontin, and fucosyltransferase 4 gene expression was significantly lower in mice groups treated with graviola extract (100 and 200 mg/kg body weight) compared to those treated with (50 mg/kg body weight), whereas expression was highest in the untreated cancerous group [178].

The chemopreventive effects of an *A. muricata* leaf ethanolic extract (AMLE) were also tested using a DMBA/croton oil-induced mice skin papillomagenesis model [179]. With the average latent period being significantly longer in the AMLE-treated group, topical application of AMLE at 100 and 300 mg/kg completely inhibited tumor development at all stages, and tumor growth from the AMLE-treated groups showed only slight hyperplasia and absence of keratin pearls and rete ridges, indicating that *A. muricata* leaves extract was able to inhibit tumor initiation as well as tumor promotion even at lower dosages [179].

*A. muricata* leaf extract (400 mg/kg) was tested for its antiproliferative and chemopreventive effects on the Ehrlich ascites carcinoma and benzo[a] pyrene-induced lung carcinoma mouse models. The results showed that the extract significantly protected against benzo[a] pyrene-induced carcinogenesis and effectively suppressed lung cancer, while in the Ehrlich ascites carcinoma model, it decreased the viable cell count and restored the hematological parameters to more or less normal levels [180].

On orthotopic tumor xenografts, graviola extract efficacy on pancreatic tumor growth was studied [112]. The pancreatic cancer cell line CD18/HPAF expressing luciferase were implanted orthotopically into the pancreas of immunodeficient mice for tumorigenic experiments. After one week, biophotonic imaging in vivo verified tumor growth in every animal, and the animals were treated with PBS alone (0 mg/kg) (control group), 50 mg/kg graviola extract, or 100 mg/kg graviola extract. Imaging was performed every two weeks to track the progression of the tumor during treatment. After 35 days of treatment, the pancreatic tumors were excised and weighed. Even though pancreatic tumors were not fully eradicated, the data indicate that tumor growth was greatly reduced in mice treated with graviola extract compared to the control group. Although all metastatic lesions were reduced in mice treated with graviola extract compared to untreated control mice, the incidence of mesenteric lymph nodes, liver, and ovary metastasis was dramatically reduced [112].

Pre-treatment with *A. muricata* at various doses (50–200 mg/kg body weight) reduces tumor incidence in DMBA-treated mice; the 200 mg *A. muricata* treatment had the greatest effect on tumor cells and reduced tumor volume [178]. In vivo studies of 1, 2-dimethyl hydrazine-induced colon cancer (Wistar albino rats) treated with *A. muricata* ethanolic leaf extract (dose of 300 mg/kg) resulted in apoptosis induction by up-regulating proapoptotic caspase-3 marker activity [181].

## 9. Clinical Studies

*A. muricata* has only been the subject of a small handful of randomized controlled trials (RCTs) that have been published, and most of them have been investigated on postresection colorectal cancer patients [182,183,184]. Twenty-eight patients with colorectal cancer participated in a randomized, double-blind, placebo-controlled trial [182,183] and they took either maltose (placebo group) or an ethanol-soluble fraction of the aqueous extract from *A. muricata* leaves (ESFAM) (supplemented group) [182,183]. Higher cytotoxicity was seen in the supplemented group compared to the placebo group in ex vivo and clinical trials [21,183]. The research confirmed that the ESFAM group’s serum selectively impeded the expansion of colorectal cancer cells while leaving healthy cells alone. Liver and renal function indices were unaffected and within a normal range following supplementation. Only a small percentage of patients (6.7%) experienced adverse effects, demonstrating that the amount administered was mostly safe and well tolerated by most of the patients [182]. There was no decrease in body weight or nutrition intake following supplementation with *A. muricata* extract, proving there was no harmful effect on compliance despite the side effects of chemotherapy [182]. However, a study with a large cohort of colorectal cancer patients and a complete investigation of the efficacy and safety of each fraction of the water extract is needed to validate this finding and recalculate the dose.

In another study, twenty outpatients with colorectal cancer who underwent primary tumor resection were split evenly between two groups: one received AML extract, and the other received maltose as a placebo before and after surgery. Overall, in the AML group, inflammation triggers anti-inflammatory mechanisms, leading to normal homeostasis [184]. The flavanes in AML have been shown to suppress interleukin-6 (IL-6) production and promote IL-2 release. IL-6 is secreted by monocytes, macrophages, and colon cancer cells, and elevated serum IL-6 levels have been associated with tumor formation, dissemination, and a poor prognosis [185]. A reduction in IL6 and an increase in IL-2 may counteract the pro-inflammatory response by encouraging the development of regulatory T cells (Tregs) and boosting IL-10 production [184]. In patients who linked lifestyle changes with the consumption of 5 g of powdered *A. muricata* leaf and seed, the malignancy was reduced with a significant decrease in colon tumor cells [186].

One clinical case report detailed the progression of metastatic breast cancer (lung and liver metastases) in a patient despite multiple cycles of treatment with anthracyclines and taxanes. Consuming about 227 g of *A. muricata* leaves decoction daily and capecitabine (2500 mg) 2 weeks on and one week off showed stability in the breast cancer patient with no side effects [187]. The liver enzymes and cancer biomarkers of the patient were found to be lowered and a PET/CT scan showed improvement and disease stabilization [187]. The patient did not suffer from graviola’s potentially fatal side effects, which can include Parkinsonism-like movement abnormalities and myeloneuropathy [187]. The necessity of retaining the natural composition of complete extracts is emphasized by the fact that isolating the “most active fraction” or single elements from whole extracts may not only compromise therapeutic efficacy but also render toxicity. In one of the trials, the researchers demonstrated how flavonoids and acetogenins work together to provide protection from the potentially lethal effects of acetogenins [127]. It was hypothesized that the flavonoid rutin was responsible for this protective effect by acting as a p-glycoprotein inducer to boost acetogenins efflux in brain [127]. In human prostate tumor xenografts, oral dosing of 100 mg/kg BW graviola leaf extract (GLE) inhibited tumor growth more effectively than flavonoid-enriched fraction (FEF), despite the larger concentrations of rutin and quercetin-3-glucoside in FEF. In contrast, despite its higher in vitro and in vivo efficacy, acetogenin-enriched fraction (AEF) caused the mice’s death owing to toxicity [127]. This revealed the significance of using leaf extract rather than isolated acetogenins [22].

In another study, some chemotherapy patients felt better while taking the *A. muricata* leaves extract supplement, and one subject reported that the mass in her abdomen was less bothersome [188]. The most often prescribed chemotherapeutic drug, cisplatin (CP), can cause significant gastrointestinal problems, and gastroprotective medicines have demonstrated partial protection against CP-induced intestinal damage [188]. The leaf extract of *A. muricata* was also reported to influence the biodistribution of the radiopharmaceutical 99mTc-DMSA [189]. This knowledge may have significant ramifications for individuals who use *A. muricata* concurrently with medical imaging [189].

Graviola has been shown to have therapeutic effects against a variety of human malignancies and disease agents in in vitro culture and preclinical animal model systems, where it has been shown to have minimal to no effect on normal cell viability while targeting the disease [21,22]. Nonetheless, more research is needed to determine the safety and tolerability of *A. muricata* leaf extract in a wider range of persons afflicted by various malignancies.

## 10. Conclusions

This current review highlights the anticancer potential and other health advantages of *A. muricata* by offering insights into its bioactive chemical components. Also, the in vitro and in vivo investigations performed to clarify the molecular mechanisms of action of its constituents have been reviewed. The plant’s acetogenins and other secondary metabolites, such as alkaloids, have been shown to inhibit cancer growth, and this potential could be fully explored. As our understanding of the molecular mechanisms of different components of graviola extract that regulate metastasis, proliferation, apoptosis, and cell signaling grows, so does the allure of the concept of employing these components in a tailored method to strengthen our arsenal against cancer. Most of the long-touted advantages have been backed by in vitro and preclinical in vivo investigations, but they still need to be verified in human clinical trials. Moreover, the toxicological profile must be recorded for increased security [177,190]. Clinical trials examining the vast pharmacological potential of *A. muricata* are another area that has been overlooked but now requires close attention. The widespread use of graviola in traditional medicine shows that, if carefully explored, these compounds could provide great benefits by making effective cancer treatments more accessible and affordable.

## Figures and Tables

**Table 1:** Ethnomedical uses of *A. muricata*.

Ethnomedicinal Uses	Plant Parts Used	Graviola Extract/Chemical Compound	References
Insecticide	Seed, leaves, barks, stems, roots and flowers	Acetogenins	[79,80]
Parasiticide	Leaf	Ethanolic extract and its fractions, methanol extracts, and acetogenins, ethyl acetate extract	[81,82,83,84]
Hypotensive	Leaf, fruit	Aqueous extract, the alkaloids, isoquinoline, coreximine, and anomurine	[85]
Fever	Leaf	Flavonoids	[70,86]
Respiratory illness	Leaf	Essential Oil	[47,70]
Sedative	Leaf	Hydroalcoholic extract	[87]
Malaria	Seed, leaf	Ethanolic extract	[50,88]
Gastrointestinal disorders	Leaf	Ethyl acetate extract	[70,89]
Liver, heart, and renal disorders	Fruit, Leaf	Ethyl acetate and ethanol extracts	[50,89,90,91,92,93]
Hypoglycemic	Leaf, branch	Ethanolic extract	[91,94,95]
Cancer	Leaf, fruit, stem, bark and branch	Annonaceous acetogenins, alkaloids, flavonoids, sterols, and others	Discussed in detail in the following sections

**Table 2:** *Annona muricata* induced anticancer effects against lung cancer.

Cancer Type	Models Used	Concentration	*A. muricata* Extract	Outcome	References
Lung cancer	In vitro (H1299) cell line	146 μg/mL (IC50 Value)	*A. muricata* leaf extract	Cytotoxic activity	[118]
	In vitro (A549) cell line	194 μg/mL (IC50 Value)	*A. muricata* leaf extract	Cytotoxic activity	[118]
	In vitro (A549) cell line	6 µg/mL	Green synthesis of silver nanoparticles using *A. muricata* leaves extract	Cell cycle arrest, elevated levels of apoptotic proteins, down regulation in Bcl-2 and cell cycle regulators, upregulation in apoptotic genes	[119]
	In vitro (A549) cell line	>100 ± 1 µg/mL (IC50 at 24 h treatment)	*A. muricata* Root Extract-derived Biogenic Silver Nanoparticles	Anti-proliferative activity	[120]
		>80 ± 1 µg/mL (IC50 at 48 h treatment)			
		>70 ± 3 µg/mL (IC50 at 72 h treatment)			

**Table 3:** *A. muricata* induced anticancer effects against prostate cancer.

Cancer Type	Models Used	Concentration	*Annona muricata* Extract	Outcome	References
Prostate cancer	In vitro PC-3 cell line	80 μg/mL (IC50 Value)	*A. muricata* leaf extract	Cytotoxic activity	[118]
	In vitro PNT1-A cell line	375.68 μg/mL (IC50 Value)	Green synthesized *A. muricata* fruit extract silver nanoparticles	Cytotoxic activity, upregulation of *CASP9* at 1.37-fold, upregulation of CXCL1 (7.17-fold), CXCR2 down regulated (0.66-fold)	[128]
		112.29 μg/mL (IC50 Value)	Green synthesized *A. muricata* leaf extract silver nanoparticles	Cytotoxic activity, upregulation of *CASP9* at 16.78-fold, upregulation of CXCL1 (85.96-fold), CXCR2 up regulated	
	In vitro PC-3 cell line	48.17 μg/mL (IC50 Value)	Green synthesized *A. muricata* fruit extract silver nanoparticles	Cytotoxic activity	
		47.58 μg/mL (IC50 Value)	Green synthesized *A. muricata* leaf extract silver nanoparticles	Cytotoxic activity	
	In vivo	90:22.5:100 mg/kg^−1^	Combination of Arginine (L-ARG), Monosodium Glutamate (MSG), Ethanolic leaves extract	Induced annexin 7 gene mutation in malignant prostatic hyperplasia-induced male Wistar rats	[129]
	In vivo	50 μM	Combination of hexane fraction (*A. muricata* seeds) and Finasteride	Prostate weight reduction in BPH induced rats	[103]
	In vitro DU-145 cell line	0.1 ± 0.07 μM (IC50 Value)	Annonacin	Cytotoxic effect	[130]
		0.5 μM		Angiogenesis	
		55.501 ± 0.55 μg/mL (IC50 Value)	Ethyl acetate bark extract	Cytotoxic effect	
		50 μg/mL		Cell migration	
		100 μg/mL		Angiogenesis	
		0.0002 μg/mL	Docetaxel in combination with 100 μg/mL Ethyl acetate bark extract	Elevated intracellular ROS, mitochondrial membrane depolarization, 3/7 caspase activation.	

**Table 4:** *Annona muricata* induced anticancer effects against breast cancer.

Cancer Type	Models used	Concentration	*Annona muricata* Extract	Outcome	References
Breast cancer	In vitro (MCF-7) cell line	2.996 µg/mL (IC50 Value)	Green synthesis of silver nanoparticles using *A. muricata* leaves extract	Antiproliferative activity	[139]
		3.109 µg/mL (IC50 Value)	Green synthesis of silver nanoparticles using *A. muricata* peel extract		
		1278 µg/mL (IC50 Value)	*A. muricata* peel extract		
		2280 µg/mL (IC50 Value)	*A. muricata* leaf extract		
	In vitro (MDA-MB-468) cell line	1.685 µg/mL (IC50 Value)	Green synthesis of silver nanoparticles using *A. muricata* leaves extract	Antiproliferative activity	[139]
		1.910 µg/mL (IC50 Value)	Green synthesis of silver nanoparticles using *A. muricata* peel extract		
		264.9 µg/mL (IC50 Value)	*A. muricata* peel extract		
		776.4 µg/mL (IC50 Value)	*A. muricata* leaf extract		
	In vitro (MCF-7) cell line	12 µg/mL	Solid lipid nanoparticles of *A. muricata* fruit extract	Antiproliferative activity, Cell cycle arrest	[140]
	In vitro AMJ13 Cell line	17.34 µg/mL	Green synthesized *A. muricata* silver nanoparticles	Anti-proliferation, apoptosis, Mitochondrial membrane potential, upregulated P53, down regulated caspase-1, IL-1β, ASC protein, NLRP3 degradation, autophagy	[141]
	In vitro (MCF-7) cell line	4.75 μg/mL (IC50 Value)	Ionic liquid- *A. muricata* fruit extract	Cytotoxic activity, cell cycle arrest (G0/G1-phase), apoptosis	[142]
		85.55 µg/mL	Methanolic leaf extracts of *A. muricata*	Cytotoxic activity	[143]
		100 µg/mL		Cell cycle arrest (G1-phase), apoptosis, elevated intracellular ROS, upregulation of caspase-3	
		10 µg/mL	Annonacin	Genotoxic activity	[144]
		0, 50, 100 and 200 µg/mL	*A. muricata* leaf extract	ER-dependent mechanism of apoptosis	
	In vitro TNBC MDA-MB-231 cell line	0, 50, 100 and 200 µg/mL	*A. muricata* leaf extract	Clonogenicity, Sub-G1 cell cycle arrest, impaired cell motility and invasiveness, intrinsic apoptotic pathway	
	In vitro MCF-7 cell line	220 µg/mL (IC50 Value)	*A. muricata* leaf extract	Cytotoxic activity	[118]

**Table 5:** *Annona muricata* induced anticancer effects against colon cancer.

Cancer Type	Models Used	Concentration	*Annona muricata* Extract	Outcome	References
Colon cancer	In vitro HCT-116 cell line	1.285 µg/mL (IC50 Value)	Green synthesis of silver nanoparticles using *A. muricata* leaves extract	Antiproliferative activity	[139]
		2.004 µg/mL (IC50 Value)	Green synthesis of silver nanoparticles using *A. muricata* peel extract		
		309.3 µg/mL (IC50 Value)	*A. muricata* peel extract		
		404.8 µg/mL (IC50 Value)	*A. muricata* leaf extract		
	In vitro HCT-116 cell line	69 ± 2.0 µg/mL (IC50 at 24 h treatment)	*A. muricata* Root Extract-derived Biogenic Silver Nanoparticles	Anti-proliferative activity, cell cycle arrest, apoptosis	[120]
		44 ± 1.5 µg/mL (IC50 at 48 h treatment)			
		8.5 ± 3 µg/mL (IC50 at 72 h treatment)			

**Table 6:** *Annona muricata* induced anticancer effects against Hematological malignancies.

Cancer Type	Models Used	Concentration	*Annona muricata* Extract	Outcome	References
Hematological malignancies	In vitro k562 Cell line	28.82 µg/mL (IC50 Value)	*A. muricata* acetone leaf extract	Cytotoxic effect, G0/G1 cell cycle arrest	[158]
		32.49 µg/mL (IC50 Value)	*A. muricata* methanol leaf extract		
	In vitro THP-1 Cell line	17.34 µg/mL	Green synthesized *A. muricata* silver nanoparticles	Anti-proliferation, apoptosis, Mitochondrial membrane potential, upregulated P53, down regulated caspase-1, IL-1β, ASC protein, NLRP3 degradation, autophagy	[141]
	In vitro Raji (Human Burkitt’s Lymphoma B-lymphoblastoid) cell line	19.1 ± 1.4 µg/mL (IC50 Value)	Hexane leaf extract	Cytotoxic effect	[53]
		4.7 ± 1.4 µg/mL (IC50 Value)	Dichloromethane leaves extract		
		80.4 ± 1.2 µg/mL (IC50 Value)	Methanol leaves extract		
		193.8 ± 1.9 µg/mL (IC50 Value)	Ethanol leaves extract		
		73.1 ± 1.4 µg/mL (IC50 Value)	Aqueous leaves extract		
		385.2 ± 1.7 µg/mL (IC50 Value)	Aqueous fruit extract		
		2.89 ± 1.3 µg/mL (IC50 Value)	Annonacin		

**Table 7:** *Annona muricata* induced anticancer effects against liver cancer.

Cancer Type	Models Used	Concentration	*Annona muricata* Extract	Outcome	References
Liver cancer	In vitro HepG-2 cell line	62.699 µg/mL (IC50 Value)	Aqueous fruit extract	Anti-proliferative activity	[74]
		63.710 µg/mL (IC50 Value)	Chloroform fruit extract		
		20.617 µg/mL (IC50 Value)	Ethyl acetate fruit extract		
		44.553 µg/mL (IC50 Value)	Hexane fruit extract		
		13.104 µg/mL (IC50 Value)	Methanol fruit extract		
		13.104 µǥ/mL and 1000 µǥ/mL (IC50 and maximum concentration)	Methanol fruit extract	Nuclear condensation	
		(13.104 µǥ/mL and 1000 µǥ/mL) (IC50 and maximum concentration)	Methanol fruit extract	Apoptosis	
	In vitro Hep2 cell line	17.98 µg/mL (IC50 Value)	Green synthesized *A. muricata* fruit pulp mediated gold nanoparticles	Antiproliferative activity	[76]
		13.08 µg/mL (IC50 Value)	Green synthesized *A. muricata* fruit peel mediated gold nanoparticles		
	In vivo Mono sodium glutamate-induced hepatic injury in rats	200 mg/kg (Body Weight)	*A. muricata extract*	Upregulated SIRT1 expression, down regulated FAS, ROS, IL-6, P53, Caspase-3, Bax levels when compared to Mono Sodium Glutamate induced rats	[92]
	In vitro HepG2 cell line	274.9 ± 8.3 µg/mL (IC50 Value)	*A. muricata* fruit extract (AMF)	Cytotoxic activity	[161]
		53.7 ± 4.3 µg/mL (IC50 Value)	Chloroform fraction of AMF	Cytotoxic activity, Significant inhibition of cell migration, apoptosis, G_0_/G_1_ phase cell cycle arrest	
		55 µg/mL			
		341.4 ± 6.7 µg/mL (IC50 Value)	Ethyl acetate fraction of AMF	Cytotoxic activity	
		928.8 ± 10.5 µg/mL (IC50 Value)	Petroleum ether fraction of AMF	Cytotoxic activity	

**Table 8:** *Annona muricata* induced anticancer effects against cervical cancer.

Cancer Type	Models Used	Concentration	*Annona muricata* Extract	Outcome	References
Cervical cancer	In vitro HeLa cell line	100 μg/mL (IC50 Value)	*A. muricata* leaf extract	Cytotoxic activity	[118]
	In vitro HeLa cell line	>70 ± 1.0 µg/mL (IC50 at 24 h treatment)	*A. muricata* Root Extract-derived Biogenic Silver Nanoparticles	Anti-proliferative activity	[120]
		>70 ± 2 µg/mL (IC50 at 48 h treatment)			
		60 ± 2.0 µg/mL (IC50 at 72 h treatment)			
	In vitro HeLa cell line	38.58 μg/mL (IC50 Value)	Green synthesized *A. muricata* fruit extract silver nanoparticles	Cytotoxic activity, CXCL1 down regulated (1.53-fold), CXCR2 down regulated (0.64-fold)	[128]
		57.63 μg/mL (IC50 Value)	Green synthesized *A. muricata* leaf extract silver nanoparticles	Cytotoxic activity, CXCL1 was down regulated (1.52-fold), CXCR2 down regulated (1.12-fold)	
	In vitro HeLa cell line	5.91 μg/mL (IC50 Value)	Ethanolic leaf extract	Cytotoxic activity	[163]
		7.56 μg/mL (IC50 Value)	Ethyl acetate leaf extract		
		8.39 μg/mL (IC50 Value)	Hexane leaf extract		
